# Crystal structure of the monoglycidyl ether of isoeugenol

**DOI:** 10.1107/S2056989022009264

**Published:** 2022-09-27

**Authors:** Hélène Cattey, Gilles Boni, Sylvie Pourchet, Laurent Plasseraud

**Affiliations:** a ICMUB CNRS UMR 6302, Université de Bourgogne Franche-Comté, Faculté des Sciences, 9 avenue Alain Savary, 21000 Dijon, France; Universidad de Los Andes, Venezuela

**Keywords:** crystal structure, oxirane, phenyl­propene, bio-based mol­ecule, isoeugenol deriv­ative

## Abstract

The title compound, C_13_H_16_O_3_ [**GE-isoEu**; systematic name: 2-({2-meth­oxy-4-[(*E*)-1-propen-1-yl]phen­oxy}meth­yl)oxirane], which crystallizes in the triclinic *P*  space group, was synthesized in one step from *iso-*eugenol, a bio-based phenyl­propanoid, with an excess of epi­chloro­hydrin. Colourless prismatic crystals suitable for X-ray diffraction were obtained from a mixture of ethyl acetate and cyclo­hexane, during purification by column chromatography on silica gel. **GE-isoEu**, which corresponds to the *trans* isomer of the monoglycidyl ether of *iso-*eugenol, is based on a 1,2,4-tris­ubstituted benzene ring by diglycidyl ether, meth­oxy and 1-(*E*)-propenyl groups, respectively. In the crystal, mol­ecules are organized through offset *π*-stacking inter­actions. Chemically, **GE-isoEu** constitutes an inter­mediate in the synthesis protocol of 2-[3-meth­oxy-4-(2-oxiranylmeth­oxy)phen­yl]-3-methyl­oxirane (**GEEp-isoEu**), a di­epoxy­dized monomer used in the manufacturing of thermosetting resins and intended for the elaboration of bio-composites.

## Abstract 2

The crystal structure of the *trans* isomer of monoglycidyl ether of *iso-*eugenol [**GE-isoEu**; systematic name 2-({2-meth­oxy-4-[(*E*)-1-propen-1-yl]phen­oxy}meth­yl)oxirane] was determined. From a supra­molecular point of view, mol­ecules of **GE-isoEu** are stacked through offset π-stacking inter­actions involving the aromatic rings.

## 1. Chemical context

The bis­phenol A diglycidyl ether mol­ecule, also called BADGE or DGEBA, is the main building block used for the formulation of commercial ep­oxy resins (Mohan, 2013 ▸). Synthetically, this reagent is directly produced from 2,2-bis­(4-hy­droxy­phen­yl)propane (bis­phenol A), derived from petroleum resources (phenol) and recognized as being an endocrine disruptor (Fenichel *et al.*, 2013 ▸). With the aim of designing more sustainable synthetic routes and alternatives to fossil resources, some of the mol­ecules derived from biomass and in particular phenyl­propano­ids isolated from the fragmentation of lignin, are considered as potential building blocks to replace bis­phenol A and its derivatives (Auvergne *et al.*, 2014 ▸). This is particularly the case of the *iso*-eugenol mol­ecule generally used in the composition of many perfumes and which can be also transformed into a diepoxidized monomer, 2-[3-meth­oxy-4-(2-oxiranylmeth­oxy)phen­yl]-3-methyl­oxirane (**GEEp-isoEu**), well-suited to ep­oxy thermosetting applications and exhibiting comparable thermomechanical properties to DGEBA (François *et al.*, 2016 ▸, 2017 ▸). The preparation of **GEEp-isoEu** involves a two-step synthesis *via* firstly the formation of the title compound as a synthesis inter­mediate. We report herein the X-ray crystal structure of **GE-isoEu** [systematic name: 2-({2-meth­oxy-4-[(*E*)-1-propen-1-yl]phen­oxy}meth­yl)oxirane], which crystallizes upon its purification from a mixture of cyclo­hexane and ethyl acetate. 
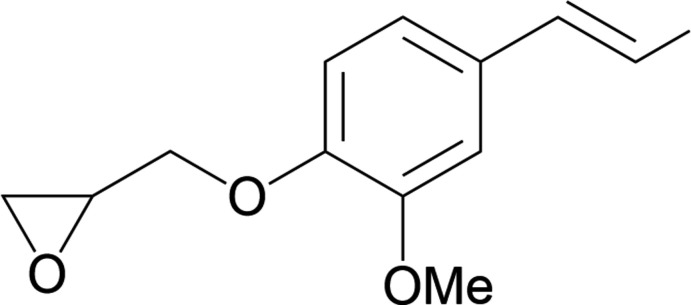


## 2. Structural commentary

**Figure 1:**
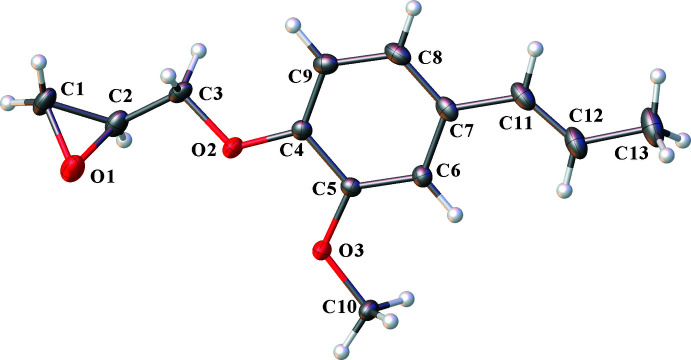
The mol­ecular structure of **GE-isoEu** with displacement ellipsoids at the 30% probability level.

The title compound exhibits an asymmetrical structure, which is depicted in Fig. 1 ▸. **GE-isoEu** comprises a benzene ring substituted by two oxygenated functional groups, glycidyl ether [–OCH_2_C_2_H_3_O] and meth­oxy [–OCH_3_], and one 1-(*E*)-propenyl side chain [–HC=CHCH_3_] located in *meta* and *para* positions to the meth­oxy and glycidyl ether functions, respectively. The aromatic ring is planar with mean bond lengths and angles of 1.392 (3) Å and 120.13 (17)°. While the –OCH_3_ and –HC=CHCH_3_ groups are in the plane of the benzene ring, –C_2_H_3_O is out of the plane with an angle of 52.83 (14)°. The oxirane ring (C1/C2/O1) of the glycidyl ether group does not undergo any disorder, in contrast to what is frequently observed for diglycidyl ether derivatives (Cho *et al.*, 1999 ▸; Flippen-Anderson & Gilardi, 1981 ▸). The double-bond distance of the 1-(*E*)-propenyl side chain was determined as 1.315 (3) Å. This length is consistent with those described in the literature for similar compounds (Stomberg *et al.*, 1993 ▸; Stomberg & Lundquist, 1995 ▸).

## 3. Supra­molecular features

**Figure 2:**
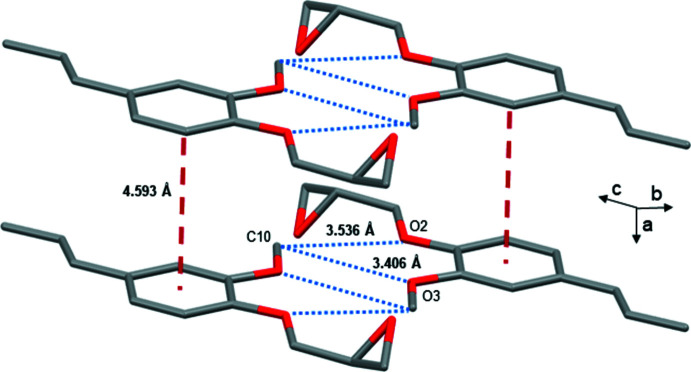
*Mercury* representation (Macrae *et al.*, 2020 ▸; colour code: C dark grey, O red; hydrogen atoms are omitted for clarity) showing inter­molecular inter­actions in the crystal structure of the title compound with hydrogen bonding (blue dotted line, C—H⋯O distance) and *π*–*π* stacking (red dotted line, centroid–centroid distance).

**Figure 3:**
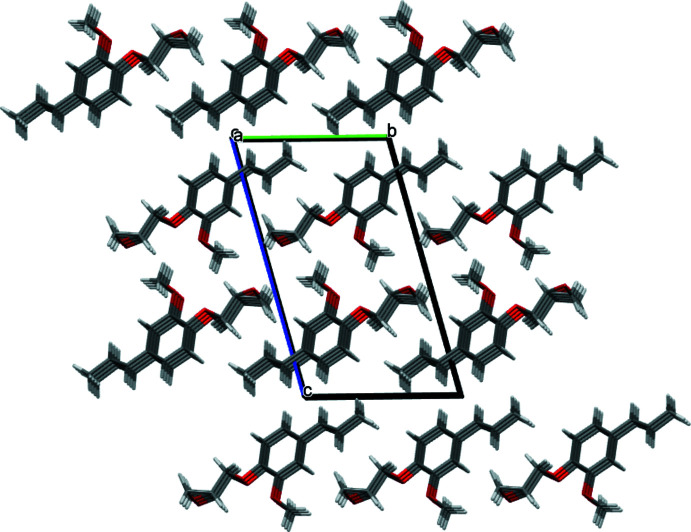
Stacking representation of **GE-isoEu** viewed down the *a* axis (colour code: C dark grey, O red, H white; *Mercury* representation; Macrae *et al.*, 2020 ▸).

The most significant supra­molecular inter­action observed in the crystal consists of offset π-stacking involving aromatic rings of **GE-isoEu** (Fig. 2 ▸ – red dotted lines). The inter­planar and the centroid-to-centroid distances between parallel mol­ecules are 3.456 (2) and 4.5931 (5) Å, respectively. The important difference between these two distances indicates that the benzene rings are strongly slipped. The slip angle (angle between the normal to the planes and the centroid–centroid vector) is 41.20° corresponding to a slippage distance of 3.025 (3) Å (these high values can nevertheless be considered as limit values). Typically, for such inter­actions, the inter­planar distance between the arene planes is found around 3.3 to 3.8 Å (Janiak, 2000 ▸). In addition, mol­ecules of **GE-isoEu** also inter­act in pairs, forming dimers, *via* non-classical inter­molecular hydrogen bonds involving the methyl groups of the meth­oxy substituents, with the oxygen atoms of the ether [C10—H10*A*⋯O2 = 3.537 (2) Å, 167°] and meth­oxy functions [C10—H10*A*⋯O3 = 3.405 (2) Å, 132°] (Fig. 2 ▸ -– blue dotted lines). These supra­molecular inter­actions result in a propagation of stacks of mol­ecules oriented along the *a*-axis (Fig. 3 ▸).

## 4. Database survey

A search in the Cambridge Structural Database (WebCSD v1.1.2, update 2022-03-05; Groom *et al.*, 2016 ▸), highlighted that, up to now, seventeen crystal structures comprising glycidyl ether-substituted phenyl ring moieties have been reported. They include (*S*)-1-(2-chloro-5-methyl­phen­oxy)-2,3-ep­oxy­propane (CIKQIZ: Bredikhin *et al.*, 2018 ▸), 2,2-bis­(3,5-di­bromo-4-hy­droxy­benzene)­propane diglycidyl ether (COMNEX: Saf’yanov *et al.*, 1984 ▸), 2,2-bis­(4-(oxiran-2-ylmeth­oxy)-3,5-di­bromo­phen­yl)propane (COMNEY: Cheban *et al.*, 1985 ▸), *rac*-1,2-ep­oxy-3-(2-meth­oxy­phen­yloxy)propane (DAXKAP: Bredikhin *et al.*, 2005 ▸), diglycidyl ether of bis­phenol A (DGEBPA: Flippen-Anderson & Gilardi, 1980 ▸; (DGEBPA01: Heinemann *et al.*, 1993 ▸; DGEBPA10: Flippen-Anderson & Gilardi, 1981 ▸), *p*-di(2,3-ep­oxy­prop­yloxy)benzene (EOXHQE: Saf’yanov *et al.*, 1977 ▸), 2,2′-[1,3-phenyl­ene-bis(oxymethyl­ene)]bis­(oxirane) (FITWOU: Bocelli & Grenier-Loustalot, 1987 ▸), 1,2-ep­oxy-3-(2-cyano­phen­oxy)propane (JESHOF: Bredikhin, *et al.*, 2006 ▸), 2-[(4-{3-[4-(oxiran-2-ylmeth­oxy)phen­yl]tri­cyclo­[3.3.1.13,7]decan-1-yl}phen­oxy)meth­yl]oxirane (LANRUQ: Wang *et al.*, 2017 ▸), 2-(4-{4-[4-(oxiran-2-ylmeth­oxy)phen­oxy]phen­yl}phen­oxy­meth­yl)oxi­rane (LAQTII: Song *et al.*, 2012 ▸), 10-[2,5-bis­(2,3-ep­oxy-1-prop­oxy)phen­yl]-9-oxa-10-phosphaphenanthren-10-one (LIPSOS: Cho *et al.*, 1999 ▸), 3,7-dimeth­oxy-2-[4-meth­oxy-3-(oxiran-2-ylmeth­oxy)phen­yl]-5-(oxiran-2-ylmeth­oxy)-4*H*-chro­men-4-one (ORASAD: Kristufek *et al.*, 2016 ▸), 2,2′-{methyl­enebis[(2,1-phenyl­ene)oxymethyl­ene]}bis­(oxirane) [PALQUS: Liu *et al.*, 2021 ▸), 2-(*N*-meth­oxy­ethanimido­yl)-5-(oxiran-2-ylmeth­oxy)benzo­nitrile (SIJZIW: Gong *et al.*, 2013 ▸); 2-({3-meth­oxy-4-[(oxiran-2-yl)meth­oxy]phen­yl}meth­yl)oxi­rane (WASCIF: Vigier *et al.*, 2017 ▸), 4-(oxiran-2-ylmeth­oxy)benzoic acid (ZEPYUQ: Obreza & Perdih, 2012 ▸). To the best of our knowledge, the structure of **GE-isoEu** based on a benzene ring tri-substituted by glycidyl ether, meth­oxy and 1-propenyl functions is new. In terms of application, several of these compounds are targeted as synthesis inter­mediates or precursors devoted to the formulation of thermosetting resins. The polymerization process involves the ep­oxy rings of mol­ecules and occurs in the presence of hardeners (such as amines or acid anhydrides), leading to the cross-linking of infusible polymer networks.

## 5. Synthesis and crystallization

**Figure 4:**
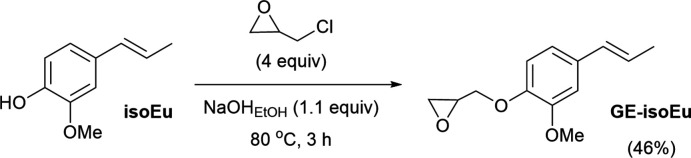
Synthesis protocol and reaction conditions leading to **GE-isoEu**.

The title compound was prepared in one step from a commercial source of natural isoeugenol (mixture of *cis*/*trans*, 99% purity, Sigma-Aldrich) according to a previously reported protocol (François *et al.*, 2016 ▸). The details of the synthesis of the title compound are summarized in Fig. 4 ▸. *iso*-Eugenol (10.0 g, 60.9 mmol) was added to an ethano­lic sodium hydroxide solution (2.7 g, 66.6 mmol of NaOH in 30 mL of EtOH), then followed by the addition of an excess of epi­chloro­hydrin (19.1 mL, 22.5 g, 243.6 mmol). The reaction mixture was heated at 353 K over 3 h under constant stirring. 70 mL of toluene were then added at room temperature. After cannula filtration, the filtrate was washed with distilled water (3 × 20 mL) and brine (1 × 20 mL), and finally dried over anhydrous MgSO_4_. After complete evaporation of the solvent under vacuum, the residue was then further purified by column chromatography using a mixture of cyclo­hexa­ne/ethyl acetate (3:1, *v*/*v*) as eluent to give a white solid characterized as the monoglycidyl ether of *iso-*eugenol (6.2 g, yield 46%). Crystals of the *trans* isomer of **GE-isoEu** suitable for X-ray analysis were obtained during the purification by column chromatography by evaporation of the eluting solvents at room temperature. ^1^H NMR (300.1 MHz, CDCl_3_): 6.90–6.82 (*m*, 3H, ar­yl), 6.08 (*dq*, *J* = 6.6 and 15.7 Hz, 1H, C*H*-propen­yl), 6.33 (*dd*, *J* =1.6 and 15.7 Hz, 1H, C*H*-propen­yl), 4.21 (*dd*, *J* = 11.4 and 3.6 Hz, 1H, C*H*_2_O), 4.03 (*dd*, *J* = 11.4 and 5.5 Hz, 1H, C*H*_2_O), 3.88 (*s*, 3H, OC*H*_3_); 3.38 (*m*, 1H, C*H-*oxirane), 2.89 (*dd*, *J* = 5.1 and 4.2 Hz, 1H, C*H*_2_ *-*oxirane), 2.73 (*dd*, *J* = 5.0 and 2.7 Hz, 1H, C*H*_2_ *-*oxirane), *δ* 1.86 (*dd*, *J* = 1.6 and 6.6 Hz, 3H, C*H*_3_); ^13^C{^1^H} NMR (75.4 MHz, CDCl_3_) *δ* 149.6, 147.1, 132.2, 130.5, 124.2, 118.6, 114.2, 109.1, 70.3, 55.8, 50.2, 45.0, 18.4; IR (ATR): 3023, 3000, 2957, 2937, 2913, 2878, 2842, 1601, 1582, 1509, 1464, 1450, 1419, 1259, 1226, 1195, 1158, 1135, 1024, 961, 908, 857, 805, 778, 759, 734, 621, 612, 569, 464 cm^−1^. Analysis calculated for C_13_H_16_O_3_: C, 69.67; H, 6.11. Found: C, 70.28; H, 7.57.

## 6. Refinement details

Crystal data, data collection and structure refinement details are summarized in Table 1 ▸. All H atoms on carbon and oxygen atoms were placed at calculated positions using a riding model with C—H = 0.95 Å (aromatic) or 0.99 Å (methyl­ene group) with *U*_iso_(H) = 1.2*U*_eq_ and C—H = 0.98 Å (methyl group) with *U*_iso_(H) = 1.5*U*_eq_(H).

## Supplementary Material

Crystal structure: contains datablock(s) I. DOI: 10.1107/S2056989022009264/dj2051sup1.cif

Structure factors: contains datablock(s) I. DOI: 10.1107/S2056989022009264/dj2051Isup2.hkl

Supporting information file. DOI: 10.1107/S2056989022009264/dj2051Isup3.cml

CCDC reference: 2208338

Additional supporting information:  crystallographic information; 3D view; checkCIF report

## Figures and Tables

**Table 1:** Experimental details

Crystal data	
Chemical formula	C_13_H_16_O_3_
*M*_r_	220.26
Crystal system, space group	Triclinic, *P* 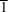
Temperature (K)	150
*a*, *b*, *c* (Å)	4.5931 (5), 8.9134 (9), 15.0764 (18)
α, β, γ (°)	74.808 (3), 85.309 (4), 77.430 (3)
*V* (Å^3^)	581.18 (11)
*Z*	2
Radiation type	Mo *K*α
μ (mm^−1^)	0.09
Crystal size (mm)	0.5 × 0.15 × 0.15

Data collection	
Diffractometer	Bruker Kappa APEXII
Absorption correction	Multi-scan (*SADABS*; Krause *et al.*, 2015 ▸)
*T*_min_, *T*_max_	0.668, 0.746
No. of measured, independent and observed [*I* > 2σ(*I*)] reflections	17290, 2647, 1865
*R*_int_	0.038
(sin θ/λ)_max_ (Å^−1^)	0.650

Refinement	
*R*[*F* ^2^ > 2σ(*F* ^2^)], *wR*(*F* ^2^), *S*	0.049, 0.128, 1.03
No. of reflections	2647
No. of parameters	147
H-atom treatment	H-atom parameters constrained
Δρ_max_, Δρ_min_ (e Å^−3^)	0.33, −0.19

Computer programs: *APEX3* (Bruker, 2020 ▸), *SAINT* (Bruker, 2016 ▸), *SHELXT2014/5* (Sheldrick, 2015*a* ▸), *SHELXL2018/3* (Sheldrick, 2015*b* ▸) and *OLEX2* (Dolomanov *et al.*, 2009 ▸).

## supplementary crystallographic information

### Crystal data

**Table:**

C_13_H_16_O_3_	*Z* = 2
*M**_r_* = 220.26	*F*(000) = 236
Triclinic, *P*1	*D*_x_ = 1.259 Mg m^−^^3^
*a* = 4.5931 (5) Å	Mo *K*α radiation, λ = 0.71073 Å
*b* = 8.9134 (9) Å	Cell parameters from 5145 reflections
*c* = 15.0764 (18) Å	θ = 2.5–26.6°
α = 74.808 (3)°	µ = 0.09 mm^−^^1^
β = 85.309 (4)°	*T* = 150 K
γ = 77.430 (3)°	PRISM, colourless
*V* = 581.18 (11) Å^3^	0.5 × 0.15 × 0.15 mm

### Data collection

**Table:**

Bruker kappa APEXII diffractometer	2647 independent reflections
Radiation source: X-ray tube, Siemens KFF Mo 2K-180	1865 reflections with *I* > 2σ(*I*)
Graphite monochromator	*R*_int_ = 0.038
Detector resolution: 8.3 pixels mm^-1^	θ_max_ = 27.5°, θ_min_ = 2.4°
φ and ω scans	*h* = −5→5
Absorption correction: multi-scan (SADABS; Krause *et al.*, 2015)	*k* = −11→11
*T*_min_ = 0.668, *T*_max_ = 0.746	*l* = −19→19
17290 measured reflections	

### Refinement

**Table:**

Refinement on *F*^2^	Primary atom site location: structure-invariant direct methods
Least-squares matrix: full	Secondary atom site location: difference Fourier map
*R*[*F*^2^ > 2σ(*F*^2^)] = 0.049	Hydrogen site location: inferred from neighbouring sites
*wR*(*F*^2^) = 0.128	H-atom parameters constrained
*S* = 1.03	*w* = 1/[σ^2^(*F*_o_^2^) + (0.0417*P*)^2^ + 0.3763*P*] where *P* = (*F*_o_^2^ + 2*F*_c_^2^)/3
2647 reflections	(Δ/σ)_max_ < 0.001
147 parameters	Δρ_max_ = 0.33 e Å^−^^3^
0 restraints	Δρ_min_ = −0.19 e Å^−^^3^

### Special details

**Table:**

Geometry. All esds (except the esd in the dihedral angle between two l.s. planes) are estimated using the full covariance matrix. The cell esds are taken into account individually in the estimation of esds in distances, angles and torsion angles; correlations between esds in cell parameters are only used when they are defined by crystal symmetry. An approximate (isotropic) treatment of cell esds is used for estimating esds involving l.s. planes.

### Fractional atomic coordinates and isotropic or equivalent isotropic displacement parameters (Å^2^)

**Table:**

	*x*	*y*	*z*	*U*_iso_*/*U*_eq_	
O3	0.5215 (3)	0.37762 (14)	0.60436 (8)	0.0300 (3)	
O2	0.8022 (3)	0.53432 (15)	0.67363 (9)	0.0327 (3)	
O1	0.7925 (3)	0.88375 (17)	0.59690 (12)	0.0498 (4)	
C5	0.6121 (4)	0.3053 (2)	0.69197 (11)	0.0234 (4)	
C6	0.5628 (4)	0.1597 (2)	0.74236 (12)	0.0262 (4)	
H6	0.457467	0.102195	0.715929	0.031*	
C4	0.7670 (4)	0.3919 (2)	0.73067 (12)	0.0247 (4)	
C7	0.6660 (4)	0.0952 (2)	0.83221 (12)	0.0307 (4)	
C9	0.8683 (4)	0.3293 (2)	0.81886 (13)	0.0322 (4)	
H9	0.973015	0.386713	0.845569	0.039*	
C3	0.9823 (4)	0.6233 (2)	0.70261 (14)	0.0319 (4)	
H3A	1.174343	0.554383	0.725793	0.038*	
H3B	0.878503	0.670214	0.752225	0.038*	
C8	0.8175 (4)	0.1822 (2)	0.86875 (13)	0.0356 (5)	
H8	0.888622	0.140217	0.929532	0.043*	
C10	0.3618 (4)	0.2968 (2)	0.56109 (12)	0.0288 (4)	
H10A	0.312228	0.360518	0.498608	0.043*	
H10B	0.177676	0.280713	0.596581	0.043*	
H10C	0.485826	0.193544	0.558386	0.043*	
C11	0.6167 (5)	−0.0610 (2)	0.88692 (13)	0.0393 (5)	
H11	0.697432	−0.096779	0.946634	0.047*	
C2	1.0334 (4)	0.7498 (2)	0.62068 (15)	0.0376 (5)	
H2	1.143379	0.712477	0.567846	0.045*	
C12	0.4742 (5)	−0.1566 (2)	0.86288 (15)	0.0462 (6)	
H12	0.392242	−0.122483	0.803335	0.055*	
C1	1.0655 (5)	0.9043 (2)	0.62760 (18)	0.0450 (5)	
H1A	1.065235	0.921634	0.689846	0.054*	
H1B	1.195933	0.961206	0.581395	0.054*	
C13	0.4301 (6)	−0.3142 (3)	0.92108 (17)	0.0583 (7)	
H13A	0.509844	−0.396339	0.888173	0.087*	
H13B	0.216596	−0.310609	0.934385	0.087*	
H13C	0.534885	−0.338975	0.978846	0.087*	

### Atomic displacement parameters (Å^2^)

**Table:**

	*U*^11^	*U*^22^	*U*^33^	*U*^12^	*U*^13^	*U*^23^
O3	0.0366 (7)	0.0279 (7)	0.0274 (6)	−0.0150 (5)	−0.0068 (5)	−0.0015 (5)
O2	0.0337 (7)	0.0320 (7)	0.0361 (7)	−0.0159 (5)	−0.0066 (6)	−0.0057 (6)
O1	0.0354 (8)	0.0392 (9)	0.0766 (11)	−0.0119 (7)	−0.0132 (7)	−0.0106 (8)
C5	0.0197 (8)	0.0257 (9)	0.0231 (8)	−0.0015 (7)	0.0001 (6)	−0.0060 (7)
C6	0.0266 (9)	0.0240 (9)	0.0268 (9)	−0.0029 (7)	0.0019 (7)	−0.0068 (7)
C4	0.0185 (8)	0.0255 (9)	0.0290 (9)	−0.0020 (7)	0.0002 (7)	−0.0071 (7)
C7	0.0323 (10)	0.0251 (9)	0.0276 (9)	0.0058 (7)	0.0037 (7)	−0.0052 (7)
C9	0.0275 (9)	0.0362 (11)	0.0331 (10)	−0.0001 (8)	−0.0045 (8)	−0.0130 (8)
C3	0.0215 (9)	0.0350 (10)	0.0444 (11)	−0.0065 (7)	−0.0044 (8)	−0.0175 (9)
C8	0.0390 (11)	0.0352 (11)	0.0259 (9)	0.0063 (8)	−0.0046 (8)	−0.0064 (8)
C10	0.0315 (9)	0.0293 (10)	0.0288 (9)	−0.0132 (7)	−0.0015 (7)	−0.0070 (7)
C11	0.0492 (12)	0.0295 (11)	0.0281 (10)	0.0056 (9)	0.0039 (9)	−0.0005 (8)
C2	0.0257 (10)	0.0380 (11)	0.0545 (13)	−0.0114 (8)	0.0037 (9)	−0.0188 (10)
C12	0.0630 (15)	0.0281 (11)	0.0378 (12)	−0.0021 (10)	0.0079 (10)	−0.0001 (9)
C1	0.0323 (11)	0.0379 (12)	0.0731 (16)	−0.0144 (9)	−0.0054 (10)	−0.0211 (11)
C13	0.0827 (18)	0.0276 (12)	0.0526 (14)	−0.0028 (11)	0.0208 (13)	−0.0029 (10)

### Geometric parameters (Å, º)

**Table:**

O3—C5	1.364 (2)	C3—C2	1.479 (3)
O3—C10	1.427 (2)	C8—H8	0.9500
O2—C4	1.368 (2)	C10—H10A	0.9800
O2—C3	1.425 (2)	C10—H10B	0.9800
O1—C2	1.430 (2)	C10—H10C	0.9800
O1—C1	1.435 (2)	C11—H11	0.9500
C5—C6	1.377 (2)	C11—C12	1.315 (3)
C5—C4	1.408 (2)	C2—H2	1.0000
C6—H6	0.9500	C2—C1	1.448 (3)
C6—C7	1.403 (2)	C12—H12	0.9500
C4—C9	1.377 (2)	C12—C13	1.495 (3)
C7—C8	1.382 (3)	C1—H1A	0.9900
C7—C11	1.474 (3)	C1—H1B	0.9900
C9—H9	0.9500	C13—H13A	0.9800
C9—C8	1.388 (3)	C13—H13B	0.9800
C3—H3A	0.9900	C13—H13C	0.9800
C3—H3B	0.9900		
			
C5—O3—C10	117.72 (13)	O3—C10—H10C	109.5
C4—O2—C3	118.70 (14)	H10A—C10—H10B	109.5
C2—O1—C1	60.72 (12)	H10A—C10—H10C	109.5
O3—C5—C6	125.24 (15)	H10B—C10—H10C	109.5
O3—C5—C4	114.77 (15)	C7—C11—H11	116.1
C6—C5—C4	119.99 (16)	C12—C11—C7	127.7 (2)
C5—C6—H6	119.5	C12—C11—H11	116.1
C5—C6—C7	120.90 (17)	O1—C2—C3	116.11 (16)
C7—C6—H6	119.5	O1—C2—H2	115.7
O2—C4—C5	114.35 (15)	O1—C2—C1	59.82 (12)
O2—C4—C9	126.27 (16)	C3—C2—H2	115.7
C9—C4—C5	119.38 (16)	C1—C2—C3	122.05 (19)
C6—C7—C11	121.60 (18)	C1—C2—H2	115.7
C8—C7—C6	118.10 (17)	C11—C12—H12	117.2
C8—C7—C11	120.29 (17)	C11—C12—C13	125.7 (2)
C4—C9—H9	120.0	C13—C12—H12	117.2
C4—C9—C8	119.97 (18)	O1—C1—C2	59.46 (12)
C8—C9—H9	120.0	O1—C1—H1A	117.8
O2—C3—H3A	110.5	O1—C1—H1B	117.8
O2—C3—H3B	110.5	C2—C1—H1A	117.8
O2—C3—C2	106.23 (15)	C2—C1—H1B	117.8
H3A—C3—H3B	108.7	H1A—C1—H1B	115.0
C2—C3—H3A	110.5	C12—C13—H13A	109.5
C2—C3—H3B	110.5	C12—C13—H13B	109.5
C7—C8—C9	121.65 (17)	C12—C13—H13C	109.5
C7—C8—H8	119.2	H13A—C13—H13B	109.5
C9—C8—H8	119.2	H13A—C13—H13C	109.5
O3—C10—H10A	109.5	H13B—C13—H13C	109.5
O3—C10—H10B	109.5		
			
O3—C5—C6—C7	179.86 (15)	C4—O2—C3—C2	167.73 (14)
O3—C5—C4—O2	−0.3 (2)	C4—C5—C6—C7	−0.3 (2)
O3—C5—C4—C9	−179.96 (15)	C4—C9—C8—C7	0.0 (3)
O2—C4—C9—C8	−179.64 (16)	C7—C11—C12—C13	−179.97 (19)
O2—C3—C2—O1	78.43 (19)	C3—O2—C4—C5	−173.56 (14)
O2—C3—C2—C1	147.72 (17)	C3—O2—C4—C9	6.1 (2)
C5—C6—C7—C8	0.2 (2)	C3—C2—C1—O1	−103.7 (2)
C5—C6—C7—C11	−179.63 (16)	C8—C7—C11—C12	178.9 (2)
C5—C4—C9—C8	0.0 (3)	C10—O3—C5—C6	0.2 (2)
C6—C5—C4—O2	179.83 (14)	C10—O3—C5—C4	−179.68 (14)
C6—C5—C4—C9	0.2 (2)	C11—C7—C8—C9	179.78 (17)
C6—C7—C8—C9	−0.1 (3)	C1—O1—C2—C3	113.5 (2)
C6—C7—C11—C12	−1.2 (3)		
